# Quantitative Assessment of the Physiological Parameters Influencing QT Interval Response to Medication: Application of Computational Intelligence Tools

**DOI:** 10.1155/2018/3719703

**Published:** 2018-01-04

**Authors:** Sebastian Polak, Barbara Wiśniowska, Aleksander Mendyk, Adam Pacławski, Jakub Szlęk

**Affiliations:** ^1^Department of Pharmacoepidemiology and Pharmacoeconomics and Department of Social Pharmacy, Faculty of Pharmacy, Jagiellonian University Medical College, Medyczna 9 Street, 30-688 Kraków, Poland; ^2^Simcyp (a Certara Company) Limited, Blades Enterprise Centre, John Street, Sheffield S2 4SU, UK; ^3^Department of Pharmaceutical Technology and Biopharmaceutics, Jagiellonian University Medical College, Medyczna 9 St, 30-688 Kraków, Poland

## Abstract

Human heart electrophysiology is complex biological phenomenon, which is indirectly assessed by the measured ECG signal. ECG trace is further analyzed to derive interpretable surrogates including QT interval, QRS complex, PR interval, and T wave morphology. QT interval and its modification are the most commonly used surrogates of the drug triggered arrhythmia, but it is known that the QT interval itself is determined by other nondrug related parameters, physiological and pathological. In the current study, we used the computational intelligence algorithms to analyze correlations between various simulated physiological parameters and QT interval. Terfenadine given concomitantly with 8 enzymatic inhibitors was used as an example. The equation developed with the use of genetic programming technique leads to general reasoning about the changes in the prolonged QT. For small changes of the QT interval, the drug-related IKr and ICa currents inhibition potentials have major impact. The physiological parameters such as body surface area, potassium, sodium, and calcium ions concentrations are negligible. The influence of the physiological variables increases gradually with the more pronounced changes in QT. As the significant QT prolongation is associated with the drugs triggered arrhythmia risk, analysis of the role of physiological parameters influencing ECG seems to be advisable.

## 1. Introduction

Human heart electrophysiology is a complex biological phenomenon, which is indirectly assessed by the measured ECG (electrocardiography) signal and its derivatives. The latter, including QT interval, QRS complex, PR interval, and T wave morphology information, are main noninvasive clinical risk markers determining patent's cardiac risk and are widely used in clinical practice for the diagnosis of cardiac disorders. Analysis of the electrographic biomarkers modification is also important from the drug development point of view, namely, assessment of drug cardiovascular safety, as it allows for quantification of drugs' and drugs candidates' influence on the human heart electrophysiology [1]. This is possible because of the well-known correlation between the drugs triggered ionic currents disruption, ECG modification, and subsequent increase in the risk of arrhythmia occurrence [2]. Probably the best established and widely used predictor of the arrhythmia risk is the QT interval prolongation compared to the baseline (delta QT or dQT) [3]. Despite the criticism that the specificity of this proarrhythmia risk surrogate is not ideal, it is still commonly used in the drug development process as well as in the clinic.

The surface ECG is a representation of the electrical activity of cardiomyocytes. The QT interval reflects the ventricular action potential duration (APD) which is determined by the flow of ionic currents across the cell membrane. The ionic currents underlying ventricular depolarization and repolarization can be disrupted by many drugs that block ion channels or ion channels trafficking. Although inhibition of the hERG channel (human ether-a-go-go-related gene) regulating the major repolarizing current in the heart, IKr (delayed inward potassium current), is the most common mechanism of QT prolongation [4, 5], it can also be caused by the drug triggered inhibition of other channels, that is, potassium (Kv7.1), sodium (Nav1.5), or calcium (Cav1.2) [6–9].

It is also known that, apart from the drug of interest, there are other parameters influencing QT interval duration. They can be divided into external (e.g., other medications taken concomitantly, which may potentiate cardiac risk by influencing the pharmacokinetics (PK) of QT-prolonging drug leading to the increase in its concentration or by the additive impact on ion channels), and internal (physiological and pathological) parameters.

The current study aimed to analyze the data from multiple virtual clinical trials simulated with the use of the biophysically detailed model of human cardiac cells physiology. The endpoint of interest was QT interval length, and the analyzed independent parameters covered external and internal parameters.

## 2. Materials and Methods

### 2.1. Data Set

Population of virtual patients exposed to terfenadine alone or in combination with various metabolic inhibitors (clarithromycin, erythromycin, itraconazole, ketoconazole, fluconazole, fluoxetine, and paroxetine) during the simulated clinical trials was used to analyze factors influencing the observed inter-individual variability [10–16]. Simcyp (version 14.1) platform was used for the PK simulations. Electrophysiological response to drug was simulated in ten Tusscher-Noble-Noble-Panfilov human ventricular cardiomyocyte model [17] implemented in Cardiac Safety Simulator™ (CSS v2.0, Certara USA, Inc.). The modeling procedure was described in detail in the recently published paper [18]. In brief, seven clinical studies focused on the electrophysiological consequences of the drug-drug interactions of terfenadine were mimicked in silico with the use of mechanistic models describing drugs pharmacokinetics and pharmacodynamics (PD). The inhibitors interacting with terfenadine covered strong, moderate, and weak inhibitors of CYP3A4-dependent terfenadine metabolism and strong CYP2D6 inhibitors. The perpetrators have diverse propensity to block hERG channel and are associated with QT prolongation and TdP risk. Fluoxetine and paroxetine are also known to block other ionic currents influencing cardiomyocyte electrophysiology, that is, ICaL (late calcium current, Cav1.2) and ICaL (late calcium current, Cav1.2) with INa (peak sodium current, Nav1.5), respectively. The endpoint of interest was QTc modification: a QT interval prolongation (as compared against a baseline), corrected for the heart rate according to the Fridericia equation [19]. Developed PK model enabled both generation of individual patient data (drugs' time-concentration profiles with corresponding values of physiological parameters influencing PK, e.g., CYP abundance) and simulation of individual pseudoECG signals dependent on i.a. age, gender, and heart parameters.

A set of 48 factors influencing the obtained differences in QTc values were analyzed. This included human related parameters (demographic, anatomical, and physiological), drug-related parameters (inhibition values of four main cardiac ionic currents), and study-dependent parameters (time of the day when the drug was taken). All of the above-mentioned parameters were generated with the use of Simcyp Simulator. The final data set consisted of 10,360 records representing QT interval durations for 63 patients taking terfenadine alone or with the concomitant drug in different time points of clinical studies [18]. The final data set consisted of 10,360 records and its summary is shown in Table 1.

Then the data set was preprocessed to overcome several potential pitfalls:

- data set was split according to the 8-fold cross-validation scheme, in each fold the data belonging to the particular metabolic inhibitors was excluded; as a consequence the developed model was forced to predict the dQTc of the unknown combination of the terfenadine-drug interaction (noted as “8cv”), which mimics the real-life application for the unknown combination,
- noise addition to prevent models from overfitting,
- noised data records were produced numerically with ±5% amplitude for each variable value and two times more records number,
- linear scaling of the data in the range of 〈0.1; 0.9〉 was applied to match nonlinear activation functions of ANNs (Artificial Neural Networks).

### 2.2. Modeling

**Figure 1:**
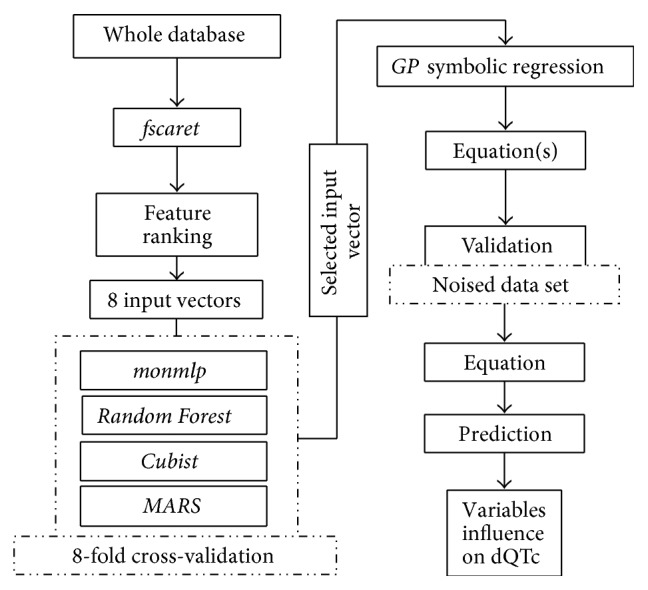
Workflow diagram presenting modeling methodology.

Modeling was carried out with the use of computational intelligence tools available as packages in the Open Source statistical environment *R* [20], namely,* fscaret* [21],* monmlp* (Monotonic Artificial Neural Networks) [22],* Cubist* [23],* randomForest* (RF, Random Forest) [24],* earth* (MARS, Multivariate Adaptive Regression Splines) [25],* rgp* (Genetic Programming and Symbolic Regression) [26], and* nloptr* [27]. All models were of multiple-input-single-output (MISO) type. The whole procedure was carried out in the following steps (Figure 1):

- reduction of the input vector based on feature ranking produced by* fscaret*,
- selection of the input vector yielding the lowest error among four types of models, namely: monmlp, Cubist, RF, and MARS,
- development of the mathematical equations with the use of the GP and symbolic regression based on the selected input vector,
- selection of the mathematical equation, optimization of its parameters and analysis of its variables on the differences in QTc values to create general conclusions regarding the terfenadine-drugs interaction.

Feature ranking obtained with the use of* fscaret* package of the R environment was employed to reduce the number of variables in the data set. The main advantages of the package are the vast number of available models for feature ranking creation, automation, and models verification based on the results obtained in earlier research, where the number of input variables was successfully reduced to 2% or 5% of the original vector [28, 29]. The* fscaret* work cycle involves training models, scaling each one according to the global performance, namely, mean squared error (MSE) or root mean squared error (RMSE), and summarizing results into the feature ranking. The main settings of the package were as follows: regression mode was turned on, time-limiting option* (myTimeLimit)* of single model development was set to 12 hours, and if possible all available functions were used. After the feature ranking was produced the cut-off points, which were limiting the number of the inputs, were selected according to the criteria that the decrease of the input's importance is more than 5% of its sum.

Both linear and nonlinear methods such as Cubist, monmlp, RF, and MARS were selected for screening the input vector, mainly due to their low computational cost and effective generalization ability in the regression problems.

An *R* environment tree based modeling approach utilized for the study was Cubist [23]. As a result of its application, the tree which consists of a set of linear models for each node is obtained. On a given training data a tree is generated which combines IF-THEN rules and linear regression models [30]. A maximum number of rules was fixed at 100, and the number of committees varied from 1 to 100.

Another tree based algorithm used was a Random Forest (RF) [31]. It is an ensemble learning method where multiple tree predictors are built on a randomly sampled vector of variables, and then they are merged to form one model. The sample distribution is kept same for all the trees [24]. The number of terminal nodes and trees in the single model varied from 10 to 1000. Random variables sampled for each tree were established from one to the maximum number of variables in an input vector.

Monotonic multilayer perceptrons (monmlp [22]) are generalized feed forward neural networks which are trained using nonlinear Newtonian-type minimization algorithm. The advantage of monmlp package is its ability to build models aggregated in expert committees. All created models were composed of two hidden layers, the number of neurons ranged from 4 to 50, and all models consisted of 10 ensemble networks. As a transition function hiperbolic tangent and linear function were applied to hidden and output layer, respectively. The number of iterations varied from 10 to 1000.

Multivariate adaptive regression splines (MARS) is an analysis introduced by Friedman in 1991 [32]. The model is a weighted sum of constant and basic functions multiplied by coefficients. The basic function in MARS is the hinge function. The function max⁡(0, *X* − constant) returns value *X* − const if *X* is greater than a constant, or 0 otherwise. The model is built in two steps. First, the model development starts from a single intercept and is extended iteratively by adding pairs of hinge functions. This process leads to model overfitting. Then the basic functions are removed from the model to improve generalization ability of the final model [33].* Earth* package for R environment was used in the present work as an example of the multivariate adaptive regression splines method [25].

The goodness of fit was expressed as the normalized root mean squared error (NRMSE) (see (1)) and the coefficient of determination (*R*^2^).

Equation (1) is as follows:

(1) $$\begin{array}{c} NRMSE=\frac{\sqrt{\left(\sum_{i=1}^{n}{\left({pred}_{i}-{obs}_{i}\right)}^{2}\right)/n}}{X_{max}-X_{min}}\times 100, \end{array}$$

where obs_*i*_ and pred_*i*_ are the observed and predicted values, respectively, *i* is the data record number, *n* is the total number of records, and *X*_max_ and *X*_min_ are the maximum and minimum observed values of dQTc.

The genetic programming (GP), in opposite to other algorithms, produces fully transparent models (white-box) which can be expressed in a form of the mathematical equations. Apart from clear mathematical formulation of the model, GP also offers potential of automatic variables selection, thus further narrowing down the most important variables set obtained by* fscaret*. The GP and the symbolic regression mode available in the* rgp* package [26] of the Open Source statistical environment *R* [20] were used in order to develop equations. Two heuristic strategies available as* rgp* options were applied concurrently:

(i) makeAgeFitnessComplexityParetoGpSearchHeuristic()

(ii) makeArchiveBasedParetoTournamentSearchHeuristic()

For both strategies all parameters were set to defaults.

Genetic programming (GP) is a method of automatic computer program creation. Lisp language was chosen as the main programming language for GP, because of its highly symbol-oriented structure. Therefore the algorithm was able to manipulate symbolic expressions to find a solution based on general problem definition [34].

A symbolic regression, which is based on evolutionary algorithms, was used during the research. Symbolic regression is a process of fitting the observed data by a mathematical formula. A chromosome encodes solution, namely, mathematical equation, which is further modified using genetic algorithms operations like crossover and mutations. The* rgp* package of *R* environment was used in this work [26]. Population size was set to 100 and a total number of steps in evolution process were set to 100 million. During the evolution, after a predefined number of fitness function evaluations (1000 *∗* population size) an elite of solutions was tested with 8-fold cross-validation scheme to produce generalization error. The adjustable parameters of the equations were randomly reinitialized and fitted using multivariate optimization provided by* nloptr* package [27]. Additionally, a noised data set based on the whole database was introduced for model testing in order to determine its stability. The maximum length of a chromosome, parameter* “individualSizeLimit”* of* rgp*, which defines a maximum degree of complexity of the solution varied from 10 to 100. The goodness of fit for evolved solutions during evolution process was assessed with root mean square error (RMSE). After the modeling step the resulting equations were sorted according to their complexity, number of parameters, and the error obtained on the 8-fold and noised data. The final model was selected according to multivariate criterion encompassing minimum complexity, the number of adjustable parameters, and generalization error (RMSE).

All scripts utilized in the study are available to download from “R scripts for multivariate analysis” resource page at SourceForge.net [35].

Computations were performed on the servers arranged in the grid structure and working under Linux operating systems control.

## 3. Results and Discussion

### 3.1. Results

The first stage of the research was to reduce the input vector based on the feature ranking produced by the* fscaret* package. According to the feature ranking created by* fscaret*, eight reduced input vectors containing 9, 10, 13, 14, 18, 19, and 23 independent variables were selected to perform modeling with the use of heuristic tools, namely, monmlp, Cubist, RF, and MARS. The results of the screening are presented in Table 1. The results obtained using the computational intelligence tools had an NRMSE of 8cv in a range from 3.8% to 6.2% (Table 2). The lowest generalization error was achieved for the model developed with Cubist algorithm trained on a vector with 18 inputs. The results obtained in this step indicate that there was a good potential for implementation of GP models.

Within this list, a vector of 14 inputs was selected for GP computations as it contained all crucial parameters related to the physiology (Table 3). Therefore expert knowledge was combined with the automated feature ranking tool for final crucial variables set. Further feature elimination was performed during the GP modeling and will be described later in the text. The most important parameters for dQTc included gender, weight, body surface area, cardiac output, CYPs abundance, electrolytes concentrations, and ionic currents inhibition (Table 3).

Equation (see (2)) derived from a data set consisting of 14 inputs (Table 3) yielded a generalization error in eightfold cross-validation (NRMSE) of 3.97% and coefficient of determination (*R*^2^) of 0.923. The results were comparable to the previously developed best model (Cubist, 3.7%). Therefore it was considered as not overfitted. The equation was characterized by 4 parameters (see (2)). Moreover, during the evolution process, the GP algorithm further reduced the number of necessary variables by eliminating input numbers: 1, 11, 14, 20, 22, and 29 (Table 3). The simplified mathematical model retained eight input variables, yet its predictive performance was comparable to the more complex Cubist model.

**Figure 2:**
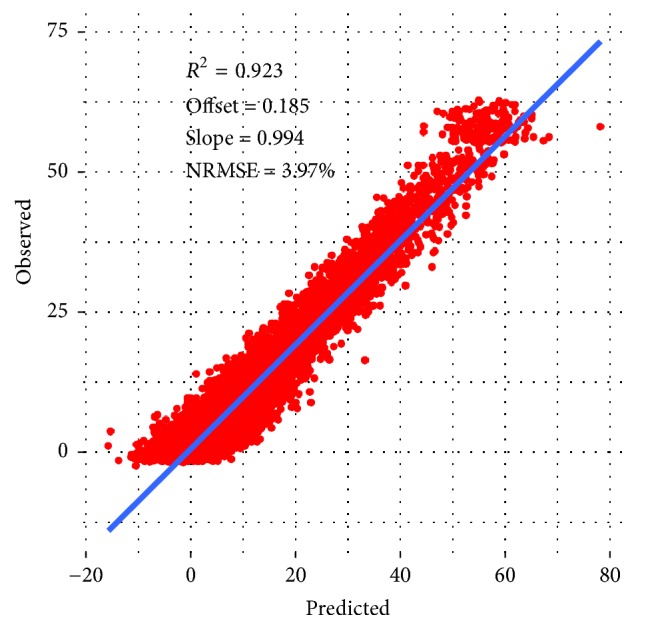
Predicted versus observed values for dQTc calculated according to (2).

Equation (2) is as follows:

(2) dQTc=sin⁡X14·X9+C2·X13·X14·X8·sin⁡X6+X14·X8+sin⁡C1·X12·X142+X11·X14+sin⁡eC4·X14·X9+X11·eeX11+C3·X11+2·sin⁡X102,

where dQTc is the difference in QTc value and *X*_6_, *X*_8_, *X*_9_, *X*_10_, *X*_11_, *X*_12_, *X*_13_, and *X*_14_ correspond to the labels in Table 2. Based on the optimization on the whole data set the adjustable parameters of (2) were as follows: *C*_1_ = −14.09525, *C*_2_ = 7.706551, *C*_3_ = 46.69071192, and *C*_4_ = 4.587024.  Figure 2 shows predicted versus observed values for dQTc calculated according to (2).

**Figure 3:**
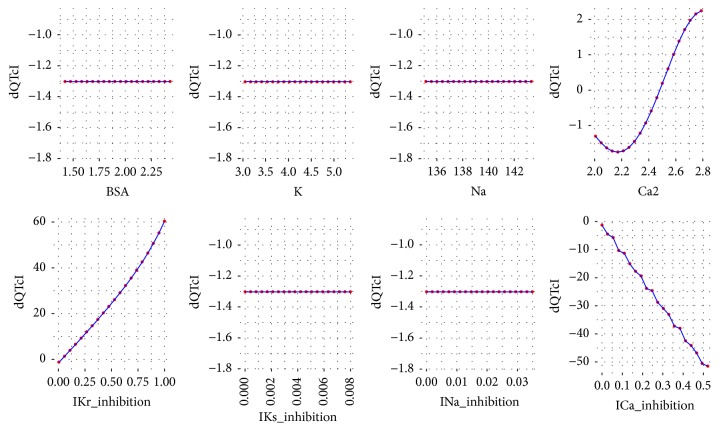
Changes in dQTc calculated according to (2), where the rest variables are of 1st quantile (0%). BSA = 1.422, K = 3.053, Na = 135.144, Ca2 = 2.007, IKr_inhibition = 0.005, IKs_inhibition = 0, INa_inhibition = 0, and ICa_inhibition = 0.

**Figure 4:**
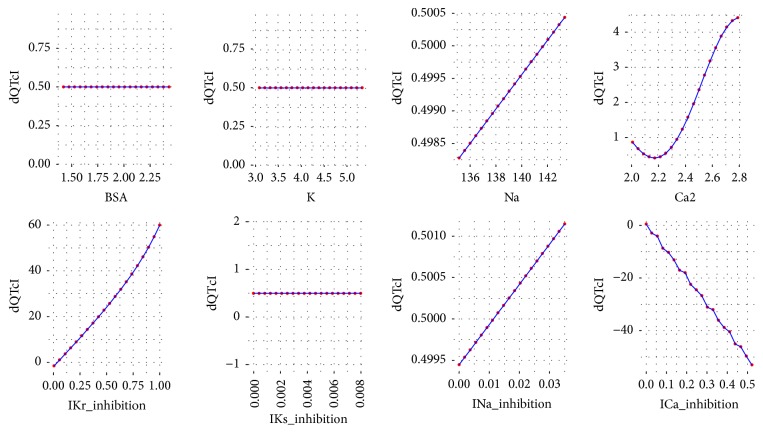
Changes in dQTc calculated according to (2), where the rest variables are of 2nd quantile (25%). BSA = 1.802, K = 4.079, Na = 139.578, Ca2 = 2.237, IKr_inhibition = 0.048, IKs_inhibition = 0, INa_inhibition = 0, and ICa_inhibition = 0.001.

**Figure 5:**
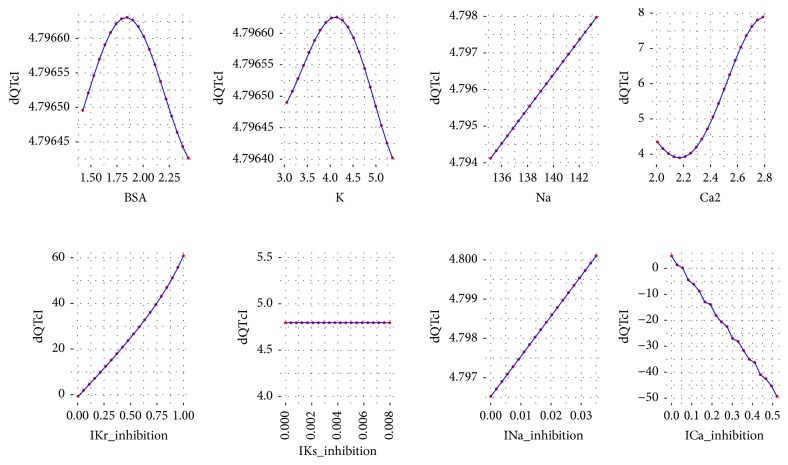
Changes in dQTc calculated according to (2), where the rest variables are of 3rd quantile (50%). BSA = 1.937, K = 4.268, Na = 140.445, Ca2 = 2.388, IKr_inhibition = 0.117, IKs_inhibition = 0, INa_inhibition = 0.001, and ICa_inhibition = 0.002.

**Figure 6:**
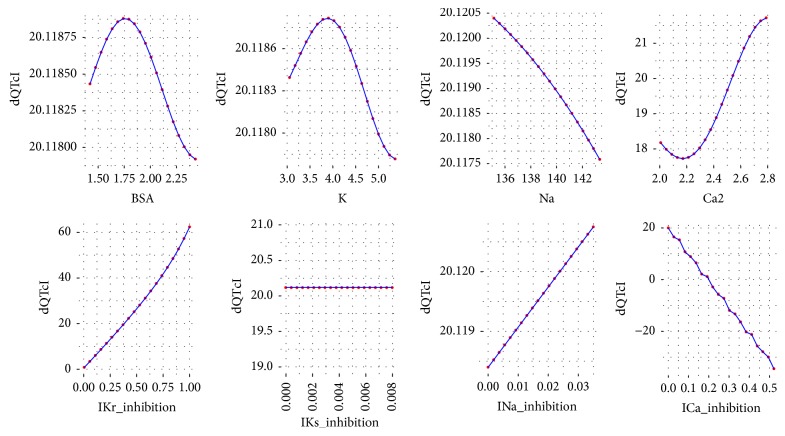
Changes in dQTc calculated according to (2), where the rest variables are of 4th quantile (75%). BSA = 2.048, K = 4.451, Na = 141.094, Ca2 = 2.546, IKr_inhibition = 0.384, IKs_inhibition = 0, INa_inhibition = 0.002, and ICa_inhibition = 0.006.

**Figure 7:**
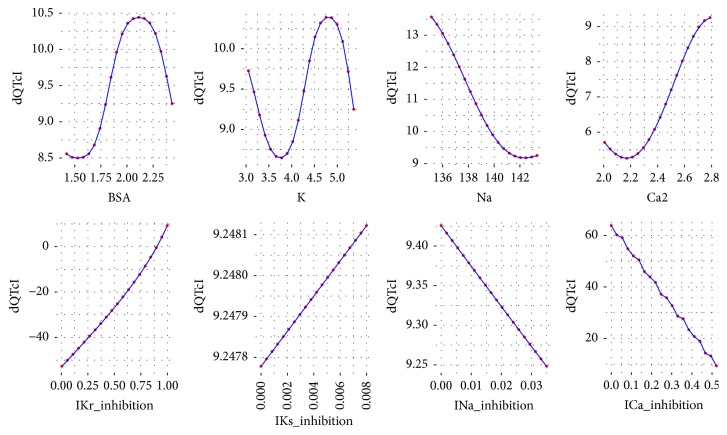
Changes in dQTc calculated according to (2), where the rest variables are of 5th quantile (100%). BSA = 2.432, K = 5.363, Na = 143.332, Ca2 = 2.789, IKr_inhibition = 1, IKs_inhibition = 0.008, INa_inhibition = 0.035, and ICa_inhibition = 0.522.

To analyze the influence of each variable of (2) on the dQTc a response analysis was performed. Each time a variable was chosen and its values were iteratively changed in a range from minimum to maximum as in the data set. The values for the remaining variables were the first, the second, the third, the fourth, and the fifth quantile (0, 25, 50, 75, and 100%). As a result, the plots were drawn for each variable versus dQTc. The plots are presented in Figures 3–7.

The model was tested on the data extrapolated for two most pronounced channel inhibition effects, namely, IKr and ICa. The inhibition values for both channels were sampled in the range 0 to 1 with an increment of 0.01 setting all the remaining variables on their median values. The resulting dQTcF values were plotted as one channel versus another matching their inhibition values.

There is a strong linear relationship between the obtained results allowing concluding on the strengths of the observed effects. The same extent of ICa inhibition produces roughly an effect of twice the magnitude of the IKr inhibition. Since both effects are contradictory, it is important information for future drug development in regard to the safety of the therapy.

### 3.2. Discussion

The applied methodology, which is an adaptation of the CRISP-DM protocol, confirmed the high quality of the developed model. Combination of feature selection tool* (fscaret)* and fast modeling techniques* (Cubist, monmlp, RF, * and* MARS)* allowed reducing the input vector by more than 50% of variables. Moreover, the obtained errors below 6.3% in the 8-fold cross-validation protocol lead to the conclusion that the developed models have not only the interpolation but also the extrapolation ability. Additionally, the equation development process and its selection assure the high quality of the GP model.

Further analysis of the equation developed by the GP technique leads to general reasoning about the changes in dQTc. It is clearly depicted that when dependent variables have small values, from the first and the second quantile (Figures 3 and 4), the differences in QTc are influenced by the IKr and ICa inhibition potentials. Moreover, the physiological parameters such as body surface area, potassium, sodium, and calcium ions concentrations have very little or none impact on the dQTc. The influence of these variables increases gradually from quantile third to fourth (Figures 5 and 6) and the highest changes in dQTc are observed when independent variables are in the fifth quantile (Figure 7). Within the data range IKs and INa inhibition currents have limited impact on the differences in QTc values (Figures 3–7). The influence of the physiological variables increases gradually with the more pronounced changes in QT. As the significant QT prolongation is associated with the drugs triggered arrhythmia risk, analysis of the role of physiological parameters influencing ECG seems to be advisable.

It is also worth noting that all the above-presented results, except the data set, were obtained with the use of the Open Source software, namely, *R* statistical environment and external packages. This study is an example of how Open Source might be exploited to create sophisticated models and modeling strategies. Use of these tools is worth consideration to provide reliable and reproducible solutions at the low cost of their development.

## 4. Conclusions

**Figure 8:**
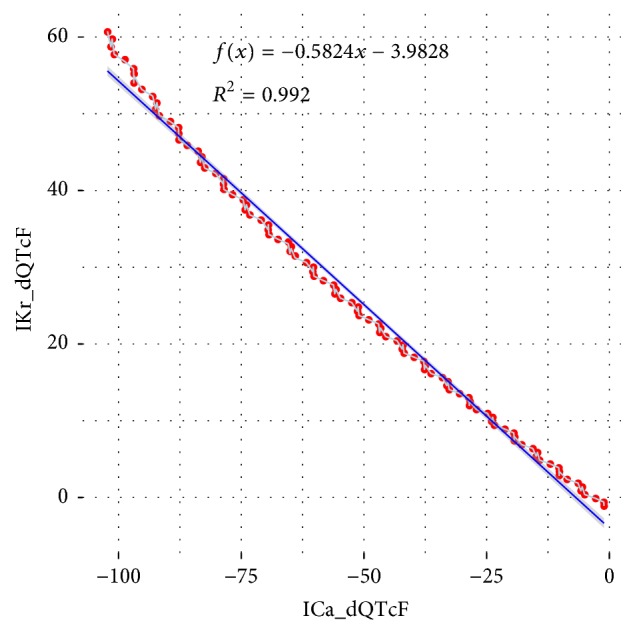
Values of dQTcF predicted for IKr versus dQTcF predicted for ICa with matching inhibition values. Based on (2).

In this work, we have shown how data processing and exploration with various computational intelligence techniques reveal hidden relationships suitable for identification of physical mechanisms relevant to the electrophysiological properties of the human cardiomyocyte. Moreover, empirical development of mathematical equations provides a convenient way to the formulation of scientific hypotheses both of the qualitative and quantitative nature. The latter was demonstrated i.a. with estimation of the relative impact of the IKr versus ICa inhibition in their antagonist influence on the QT prolongation (Figure 8).

## Figures and Tables

**Table 1:** The summary of the data set.

Input number	Labels	Min	1st quantile	Median	Mean	3rd quantile	Max
(1)	CYP1A2	0	0	0	695159	0	11963910
(2)	CYP2A6	0	0	0	0	0	0
(3)	CYP2B6	0	0	0	86155	0	9129915
(4)	CYP2C8	0	0	0	0	0	0
(5)	CYP2C9	0	0	0	347459	0	16469809
(6)	CYP2C18	0	0	0	0	0	0
(7)	CYP2C19	0	0	0	179400	0	5511004
(8)	CYP2D6	0	252231	399478	506695	676312	2185456
(9)	CYP2E1	0	0	0	0	0	0
(10)	CYP2J2	0	0	0	0	0	0
(11)	CYP3A4	1552693	5716140	8459929	9833766	12016781	32307836
(12)	CYP3A5	0	0	0	272853	0	13728653
(13)	CYP3A7	0	0	0	0	0	0
(14)	Gut_CYP2C9	8210	1430	10037.216	10089.122	10148.391	10388.032
(15)	Gut_CYP2C19	0	0	0	299	0	7330
(16)	Gut_CYP2D6	0	484.7	674.4	818.4	994.5	3156
(17)	Gut_CYP2J2	0	0	0	0	0	0
(18)	Gut_CYP3A4	11214	35540	55975	62911	80376	217447
(19)	Gut_CYP3A5	0	0	0	807.2	0	55106.1
(20)	Sex_Code	0	1	1	0.7718	1	1
(21)	Age	19	25	28	28.39	32	52
(22)	Weight	44.52	69.43	78.31	79.04	89.17	127
(23)	Height	149.4	168.6	174.3	173.6	179.7	200.5
(24)	BSA	1.422	1.802	1.937	1.929	2.048	2.432
(25)	Brain_Weight	1040	1230	1354	1388	1524	2057
(26)	Kidney_Weight	164.1	269.1	325	329.1	382.6	752
(27)	Liver_Weight	1052	1544	1700	1740	1938	2699
(28)	BMI	16.13	22.87	26.22	26.19	28.8	45.31
(29)	Cardiac_Output	249.7	315.1	339.4	337.5	357.2	424.5
(30)	Haematocrit	32.12	38.99	41.73	41.46	43.68	51.05
(31)	HSA	35.11	42.88	45.74	45.75	48.57	58.08
(32)	AGP	0.3971	0.7137	0.8035	0.7982	0.8851	1.207
(33)	Serum_Creatinine	33.64	62.09	73.26	72.61	81.2	122.95
(34)	GFR	70.97	112.12	129.74	133.34	153.5	243.16
(35)	Renal_Function	0.59	0.92	1.079	1.089	1.271	1.87
(36)	Cardiomyocyte_area	652.5	1384.3	1701.1	1824.4	2146.8	5353.7
(37)	Cardiomyocyte_volume	1852	4494	5630	6217	7346	20339
(38)	Sarcoplasmic_reticulum_volume	111.1	269.6	337.8	373	440.7	1220.3
(39)	Capacitance	17.33	36.77	45.18	48.46	57.02	142.2
(40)	String_length	0.8772	1.1814	1.293	1.2878	1.4064	1.8619
(41)	K	3.053	4.079	4.268	4.261	4.451	5.363
(42)	Na	135.1	139.6	140.4	140.3	141.1	143.3
(43)	Ca2	2.007	2.237	2.388	2.394	2.546	2.789
(44)	IKr_inhibition	0.0047	0.0484	0.1172	0.2378	0.3841	1
(45)	IKs_inhibition	0	1*E* − 04	1*E* − 04	0.0002684	0.0003	0.008
(46)	INa_inhibition	0	0.0003	0.0007	0.001493	0.0016	0.0353
(47)	ICa_inhibition	0	0.0009	0.0022	0.01782	0.0061	0.5217
(48)	Stimulation_Period	432	735	825	836.1	925	1570

Output	dQTc	−15.707	1.713	6.591	10.572	14.69	78.142

Where CYP1A2, CYP2A6, CYP2B6, CYP2C8, CYP2C9, CYP2C18, CYP2C19, CYP2D6, CYP2E1, CYP2J2, CYP3A4, CYP3A5, CYP3A7, corresponding patients' abundance of cytochromes in the liver [pmol/mg of protein]; Gut_CYP2C9, Gut_CYP2C19, Gut_CYP2D6, Gut_CYP2J2, Gut_CYP3A4, Gut_CYP3A5, corresponding patients' abundance of cytochromes in the gut [nmol/small intestine]; Sex_Code, patients' gender [male = 0/female = 1]; Age, patients' age [years]; Weight, patients' weight [kg]; Height, patients' height [cm]; BSA, patients' body surface area [m^2^]; Brain_Weight, patients' brain weight [g]; Kidney_Weight, patients' kidney weight [g]; Liver_Weight, patients' liver weight [g]; BMI, patients' body mass index; Cardiac_Output, patients' cardiac output [L/h]; Haematocrit, patients' specific haematocrit [%]; HSA, AGP, patients' specific level of human serum albumin and alfa-acid glycoproteins in the plasma [g/L]; Serum_Creatinine, patients' specific creatinine level [*μ*mol/L]; GFR, the Glomerular Filtration Rates of the simulated individual (mL/min/1.73 m^2^); Renal_Function, the ratio of individual's GFR to that of the normal value of 120 mL/min/1.73 m^2^ for male or 130 mL/min/1.73 m^2^ for female; Cardiomyocyte_area, patients' specific area of the cardiac myocyte [*μ*m^2^]; Cardiomyocyte_volume, patients' specific volume of the cardiac myocyte [*μ*m^3^]; Sarcoplasmic_reticulum_volume, patients' specific volume of the cardiac myocyte sarcoplasmic reticulum [*μ*m^3^]; Capacitance, patients' specific cardiac myocyte electric capacitance [pF]; String_length, patients' specific thickness of the left heart wall [cm]; K, Na, Ca2, patients' specific concentration of ions in plasma [mM]; IKr_inhibition, IKs_inhibition, INa_inhibition, ICa_inhibition, patients' and drugs' specific ionic current inhibition; Stimulation_Period, time gaps between stimulaitons [ms]; dQTc, patients' QTc interval modification as compared against baseline.

**Table 2:** The results (NRMSE) of four algorithms applied on the eight input vectors. Corresponding coefficients of determination (*R*^2^) are shown in brackets.

Input vector	Cubist	monmlp	RF	MARS
9in_RMSE	3.8 (0.93)	4.0 (0.92)	6.1 (0.85)	5.8 (0.79)
10in_MSE	3.9 (0.93)	3.9 (0.93)	6.0 (0.83)	6.0 (0.77)
13in_MSE	3.9 (0.93)	3.9 (0.93)	6.0 (0.83)	5.9 (0.78)
14in_RMSE	4.0 (0.93)	3.9 (0.93)	6.1 (0.82)	5.8 (0.78)
18in_MSE	3.7 (0.94)	4.0 (0.93)	6.2 (0.80)	5.8 (0.78)
19in_RMSE	3.7 (0.94)	4.0 (0.93)	6.1 (0.81)	5.8 (0.78)
23in_MSE	3.8 (0.93)	4.0 (0.93)	6.0 (0.84)	5.8 (0.78)
23in_RMSE	3.8 (0.93)	4.0 (0.93)	6.0 (0.85)	5.8 (0.78)

**Table 3:** Input vector selected for GP modeling.

Orig input number	Equation (2) label	Label	Description
(1)	-	CYP1A2	Liver CYP1A2 abundance [pmol/mg of protein]
(11)	-	CYP3A4	Liver CYP3A4 abundance [pmol/mg of protein]
(14)	-	Gut_CYP2C9	Gut CYP2C9 abundance [pmol/mg of protein]
(20)	-	Sex_Code	Patients' gender [male/female]
(22)	-	Weight	Patients' weight [kg]
(24)	*X*6	BSA	Patients' body surface area [m^2^]
(29)	-	Cardiac_Output	Patient's cardiac output [L/h]
(41)	*X*8	K	Patients' plasma potassium concentration [mM]
(42)	*X*9	Na	Patients' plasma sodium concentration [mM]
(43)	*X*10	Ca2	Patients' plasma calcium concentration [mM]
(44)	*X*11	IKr_inhibition	Patients' and drugs' specific IKr current inhibition [0–100%]
(45)	*X*12	IKs_inhibition	Patients' and drugs' specific IKs current inhibition [0–100%]
(46)	*X*13	INa_inhibition	Patients' and drugs' specific INa current inhibition [0–100%]
(47)	*X*14	ICa_inhibition	Patients' and drugs' specific ICa current inhibition [0–100%]
(49)	dQTc	dQTc	Output (QTc interval modification as compared to baseline)
